# Isolation, molecular identification, and probiotic potential of *Lactiplantibacillus plantarum* from feces of captive Asian elephants (*Elephas maximus*)

**DOI:** 10.14202/vetworld.2026.539-553

**Published:** 2026-02-10

**Authors:** Mariya Sewaka, Rachadaporn Srisamut, Patarapol Maneeorn, Wipawee Saengsoi, Dhiravit Chantip, Sirisak Cheechang, Sulaiman Madyod, Wiruntita Bohman

**Affiliations:** 1 Department of Biomedical Sciences, Faculty of Veterinary Science, Rajamangala University of Technology Srivijaya, Thung Yai, Nakhon Si Thammarat, Thailand; 2 Department of Veterinary Clinical Sciences, Faculty of Veterinary Science, Rajamangala University of Technology Srivijaya, Thung Yai, Nakhon Si Thammarat, Thailand; 3 Department of National Parks, Wildlife and Plant Conservation, Bangkok, Thailand

**Keywords:** *16S rRNA* gene, Asian elephant, gut microbiota, lactic acid bacteria, *Lactiplantibacillus plantarum*, probiotic potential, safety assessment, *in vitro* evaluation

## Abstract

**Background and Aim:**

The gastrointestinal health of Asian elephants (*Elephas maximus*) is critically dependent on hindgut microbial fermentation, yet host-specific probiotic strains derived from elephants remain poorly characterized. Although lactic acid bacteria (LAB) are widely recognized for their probiotic benefits, systematic evaluation of elephant-origin LAB, including molecular identification and safety assessment, is limited. This study aimed to isolate LAB from the feces of healthy captive Asian elephants and comprehensively evaluate their *in vitro* probiotic potential, safety profile, and molecular identity to support the development of host-adapted probiotic candidates.

**Materials and Methods:**

Fresh fecal samples were collected from 25 clinically healthy captive Asian elephants housed at four elephant camps in Krabi Province, Thailand. LAB were isolated using de Man, Rogosa, and Sharpe agar and subjected to preliminary phenotypic and biochemical characterization. Antimicrobial activity was evaluated against five pathogenic indicator bacteria using the disk diffusion method. Probiotic functional properties were assessed through acid tolerance (pH 3.0), bile salt tolerance (1%), cell surface hydrophobicity, and autoaggregation assays. Safety evaluation included hemolytic activity and antibiotic susceptibility testing. Molecular identification of selected isolates was performed using *16S rRNA* gene sequencing followed by phylogenetic analysis.

**Results:**

A total of 195 LAB isolates were recovered, of which 52 exhibited antimicrobial activity against all tested pathogens. Eleven isolates demonstrated superior probiotic attributes, with acid and bile salt survival rates ranging from 74.67%–91.67% and 75.17%–98.15%, respectively. These isolates showed strong antimicrobial activity (inhibition zones 12–15 mm), high cell surface hydrophobicity (74.03%–92.24%), and substantial autoaggregation capacity (70.60%–85.74%). All selected isolates were non-hemolytic and susceptible to clinically relevant antibiotics. Molecular analysis identified seven isolates as *Enterococcus faecalis* and four as *Lactiplantibacillus plantarum*. Among them, isolates I9, I56, and I145 (*L. plantarum*) exhibited the most consistent and robust probiotic characteristics.

**Conclusion:**

This study provides the first molecularly validated and comprehensive *in vitro* evaluation of probiotic LAB isolated from captive Asian elephants. The identified *L. plantarum* strains, particularly isolates I9, I56, and I145, demonstrated strong functional and safety profiles, supporting their potential as host-specific probiotics for improving gastrointestinal health and disease management in captive and wild Asian elephants.

## INTRODUCTION

The Asian elephant (*Elephas maximus*) is a monogastric herbivore with comparatively low efficiency in volumetric digestion. Microbial fermentation and nutrient absorption occur predominantly in the hindgut, where resident microbiota are specialized in degrading fibrous plant materials [1]. This hindgut fermentation is essential for supplying metabolic energy, particularly from indigestible dietary fibers that escape enzymatic digestion in the foregut [2]. Disruption of hindgut microbial activity can markedly reduce energy availability in elephants, adversely affecting physiological functions and overall health status [3].

With advancing age, Asian elephants experience progressive wear or loss of molar teeth, which substantially reduces their capacity to masticate fibrous plant material. Impaired mechanical digestion compromises gastrointestinal efficiency and increases the risk of digestive disturbances and malnutrition [4]. Consequently, geriatric elephants are especially vulnerable to nutritional deficiencies and gastrointestinal disorders [5, 6]. In addition, a decline in gut microbiota strains capable of inhibiting pathogenic bacteria has been associated with gastrointestinal conditions such as colic and diarrhea. Dysbiosis may also adversely affect nitrogen metabolism in the hindgut, further compromising digestive health [7]. To address these challenges, probiotic supplementation with microorganisms such as *Lactobacillus acidophilus* and *Saccharomyces cerevisiae* has been proposed to enhance digestibility and dry matter intake [8], improve mineral metabolism [9], and induce beneficial shifts in fecal microflora composition [10]. Collectively, these effects contribute to improved digestive health, overall well-being, and potential longevity in elephants [11].

Lactic acid bacteria (LAB) are widely regarded as one of the most promising groups of microorganisms for probiotic applications due to their well-established safety profiles and functional efficacy in promoting gastrointestinal health [12, 13]. LAB are naturally present in fermented foods [14, 15], the gastrointestinal tracts of humans [16] and animals, as well as diverse ecological environments [17–19]. Their probiotic effects are primarily attributed to their capacity to inhibit pathogenic microorganisms through the production of organic acids, bacteriocins, and hydrogen peroxide [20, 21]. In addition, LAB contribute to host health by enhancing intestinal barrier integrity [22], modulating immune responses [23], and competing with pathogens for adhesion sites on the intestinal epithelium [24].

Probiotic supplementation with LAB not only improves growth performance and health outcomes but also supports sustainable animal production by reducing reliance on antibiotics [25]. Probiotic effects are generally strain- and host-specific, highlighting the importance of evaluating indigenous isolates from the target species to ensure optimal compatibility, efficacy, and safety [26]. Strains originating from a specific host species often confer greater benefits when administered to the same host, as they are naturally adapted to its gastrointestinal environment. Several studies have demonstrated that host-specific probiotic strains isolated from the human gastrointestinal tract, including *Lactiplantibacillus plantarum* (formerly *Lactobacillus plantarum*), *Lactobacillus rhamnosus*, and *Lactobacillus casei*, exert immunomodulatory effects, including enhanced anti-inflammatory responses in human peripheral blood mononuclear cells [27]. Similarly, *L. plantarum* isolated from healthy swine improves intestinal microbiota composition by increasing beneficial bacteria and suppressing pathogenic populations in weaning piglets [28]. In cattle, *Lactobacillus paracasei* enhances feed utilization, increases milk yield and quality, and supports gastrointestinal health [29]. Avian-derived strains, such as *L. acidophilus* and *Streptococcus faecium*, inhibit pathogenic bacterial colonization in the poultry gastrointestinal tract [30].

Recent *16S rRNA* gene sequencing studies have characterized the gut microbiota of healthy captive Asian elephants, providing baseline data for intestinal health assessment. These studies consistently report a predominance of fiber-degrading taxa, particularly Firmicutes and Bacteroidota, underscoring the critical role of microbial fermentation in elephant hindgut physiology [31]. Such baseline microbiota profiles facilitate the identification of dysbiosis and support the development of microbiome-based interventions to improve gastrointestinal health. Moreover, host-derived probiotics are increasingly recognized as advantageous for large mammals with complex gastrointestinal systems, as they may exhibit superior intestinal adaptation and colonization compared with non-host-derived commercial strains.

The presence of *L. plantarum* in the feces of captive Asian elephants has been primarily identified using biochemical approaches. These isolates demonstrated tolerance to acidic and bile conditions, surviving across a pH range of 3–9 and in the presence of 0.30% (w/v) bile salts [32].

Despite growing evidence highlighting the importance of gastrointestinal microbiota in Asian elephants, significant gaps remain in the identification and validation of host-derived probiotic candidates. Previous investigations on LAB associated with elephants have largely relied on phenotypic and biochemical characterization, with limited molecular confirmation and incomplete functional evaluation. In particular, comprehensive *in vitro* assessments of probiotic attributes, including antimicrobial activity, tolerance to acidic and bile conditions, cell surface hydrophobicity, autoaggregation capacity, and safety parameters such as hemolytic activity and antibiotic susceptibility, are scarce. Although *L. plantarum* has been reported in elephant feces, its molecularly confirmed presence based on *16S rRNA* gene analysis and its probiotic functionality in this host remain insufficiently explored. This lack of well-characterized, elephant-specific LAB strains limits the development of targeted probiotic interventions to support gastrointestinal health, especially in captive and aging elephants that are more susceptible to dysbiosis and digestive disorders.

Therefore, this study aimed to isolate LAB from the feces of healthy captive Asian elephants and to systematically evaluate their probiotic potential using standardized *in vitro* assays. The specific objectives were to assess antimicrobial activity against selected pathogenic bacteria, tolerance to acidic and bile salt conditions, cell surface hydrophobicity, and autoaggregation ability, together with safety evaluation through hemolytic activity and antibiotic susceptibility testing. In addition, molecular identification based on *16S rRNA* gene sequencing was performed to confirm the taxonomic identity of selected isolates, with particular emphasis on *L. plantarum*. This integrated approach was designed to identify robust, host-adapted probiotic candidates with potential applications in improving gastrointestinal health and overall well-being in Asian elephants.

## MATERIALS AND METHODS

### Ethical approval

This study involved the collection of freshly voided fecal samples from captive Asian elephants and *in vitro* laboratory experiments on bacterial isolates. Animals were not restrained, sedated, or subjected to invasive procedures. Samples were collected immediately after defecation during routine husbandry at four tourist elephant camps in Krabi Province, Thailand. Written permission was obtained from all participating elephant camps, and all procedures complied with institutional and international animal welfare guidelines.

### Study period and location

The study was conducted between January 2022 and May 2024. Fecal samples were collected from captive Asian elephants at four tourist elephant camps in Krabi Province, Thailand. All laboratory procedures and analyses were performed at the Faculty of Veterinary Science, Rajamangala University of Technology, Srivijaya, Thungyai, Nakhon Si Thammarat, Thailand.

### Animals, housing, and dietary management

Twenty-five Asian elephants, comprising 23 females and two males, aged 30–50 years and weighing approximately 2,000–4,000 kg, housed at a tourist elephant camp in Krabi Province, Thailand, were included in this study. All elephants were clinically healthy and had not received any medications, dietary supplements, or probiotics prior to the collection of the samples. The elephant camp maintained high hygiene standards and was situated in a forested environment that allowed free roaming, access to natural water streams for bathing, and daily physical activity. The elephants were managed under a free-ranging, non-captive system without cage confinement, which supports good physical and psychological well-being. Low stress and normal behavior indicators, including regular ear flapping and trunk swinging, were observed. The elephants were fed a diet consisting of pineapple stems, Napier grass (*Pennisetum purpureum*), and Bana grass (*P. purpureum* × *Pennisetum americanum*).

### Collection and handling of fecal samples

Fecal samples were obtained from 25 clinically healthy captive Asian elephants aged between 30 and 50 years, housed at a tourist elephant camp in Krabi Province, Thailand. Freshly voided feces were collected within 4 h of defecation in the morning. All 25 elephants were individually sampled, and fecal samples were collected once from each animal. Fecal specimens were collected in sterile plastic zip bags and immediately placed on ice for transport to the laboratory within 3 h to preserve microbial viability.

### Isolation of LAB

For LAB isolation, 10 g of each fecal sample was diluted 10-fold in sterile 0.85% normal saline solution. Subsequently, 0.1 mL aliquots of the 10^-3^ to 10^-6^ dilutions were spread onto de Man, Rogosa, and Sharpe (MRS) agar (pH 6.5; bioMérieux, Marcy l’Etoile, France). Plates were incubated at 37°C for 48 h under aerobic conditions. The number of LAB isolates recovered per elephant was comparable, with approximately 7–8 isolates obtained from each animal.

### Preliminary LAB phenotypic and biochemical characterization

Colonies exhibiting distinct morphology were selected and repeatedly subcultured to obtain pure isolates. Preliminary phenotypic characterization was conducted by incubating the pure cultures on MRS agar at 37°C for 48 h under anaerobic conditions, followed by Gram staining, spore staining, and standard biochemical tests, including catalase test, indole test, and oxidase test to confirm their identity as LAB [33].

### Antimicrobial activity screening against pathogenic bacteria

The antimicrobial activity of LAB isolated against common pathogenic bacteria was evaluated using the disk diffusion assay. In brief, LAB isolates were cultured in MRS broth (bioMérieux, Marcy l’Etoile, France) and incubated at 37°C for 72 h under anaerobic conditions. After incubation, the cultures were centrifuged at 8,000 × *g* for 10 min to separate the bacterial cells. The supernatant was collected and further sterilized by filtration through a 0.22 µm membrane filter (Sartorius, USA) to obtain the cell-free supernatant, which was used to assess antimicrobial activity.

Pathogenic indicator bacteria, including *Escherichia coli* (American Type Culture Collection [ATCC] 25922), *Salmonella enterica* serovar Typhimurium (ATCC 13311), *Pseudomonas aeruginosa* (ATCC 27853), *Staphylococcus aureus* (ATCC 25923), and *Klebsiella pneumoniae* (ATCC 700603), were cultured in tryptic soy broth (bioMérieux, Marcy l’Etoile, France) at 37°C for 24 h under aerobic conditions. The cultures were centrifuged at 8,000 × *g* for 10 min, and the bacterial pellets were washed twice with phosphate-buffered saline (PBS; pH 7.4). PBS was prepared by dissolving 8.0 g sodium chloride (NaCl; HiMedia, India), 0.2 g potassium chloride (KCl; HiMedia), 1.44 g disodium hydrogen phosphate (Na_2_HPO_4_; HiMedia), and 0.24 g potassium dihydrogen phosphate (KH_2_PO_4_; HiMedia) in distilled water, with the final volume adjusted to 1 L and the pH adjusted to 7.4 using hydrochloric acid (HCl; HiMedia) or sodium hydroxide (NaOH; HiMedia). The buffer was sterilized by autoclaving before use.

The washed cell pellets were resuspended in sterile 0.85% NaCl and adjusted to a turbidity equivalent to a 0.5 McFarland standard (approximately 10^8^ Colony-forming units [CFU]/mL) using a suspension turbidity detector (Den-1B, Biosan, Latvia). The bacterial suspensions were swabbed onto Mueller-Hinton agar plates (HiMedia, India). Sterile MRS broth without LAB was included as a negative control for the antimicrobial assay. Sterile paper disks were placed on the agar surface, and 50 µL of cell-free supernatant from each LAB isolate was applied to the disks [34]. Plates were incubated at 37°C for 24 h under aerobic conditions, after which the inhibition zone diameters surrounding the disks were measured. These measurements were used to evaluate the LAB isolates’ inhibitory potential against the tested pathogens.

### Assessment of the acid and bile salt tolerance of the LAB isolates

#### Acid tolerance assay

LAB isolates exhibiting strong inhibitory activity against pathogenic bacteria were selected for acid tolerance assessment. These isolates were cultured on MRS agar at 37°C for 48 h under anaerobic conditions, after which the bacterial suspense was adjusted to a turbidity equivalent to 0.5 McFarland standard (approximately 10^8^ CFU/mL). To evaluate acid tolerance, 100 µL of each standardized suspension was inoculated into MRS broth (bioMérieux) adjusted to pH 3.0 using 1 M HCl. Cultures were incubated at 37°C for 0 and 3 h under anaerobic conditions. At each time point, samples were spread-plated onto MRS agar and incubated at 37°C for 48 h under anaerobic conditions. The survival rates of the LAB strains were calculated using the following formula [35]:

Survival rate (%) = (Number of viable cells after exposure/Number of viable cells at time 0) × 100

#### Bile salt tolerance assay

LAB isolates that exhibited strong antagonistic activity against pathogenic bacteria were selected for bile salt tolerance evaluation. The selected isolates were cultured on MRS agar at 37°C for 48 h under anaerobic conditions. Bacterial suspensions were adjusted to a turbidity of 0.5 McFarland standard (approximately 10^8^ CFU/mL). MRS broth supplemented with 1% bile salts (Cat. No. 0194000100; Loba Chemie, India) was prepared to assess bile salt tolerance. A 100 µL (1 × 10^7^ CFU) aliquot of the adjusted LAB suspension was inoculated into the 1% bile-containing MRS broth and incubated at 37°C for 0 and 3 h under anaerobic conditions. At each time point, the samples were spread-plated onto MRS agar and incubated at 37°C for 48 h under anaerobic conditions. To assess acid tolerance, CFU were subsequently enumerated, and the survival rate was calculated using the following formula:

Survival rate (%) = (Number of viable cells after exposure / Number of viable cells at time 0) × 100

According to widely adopted probiotic evaluation criteria, strains demonstrating survival rates of ≥ 50% under acidic and bile salt conditions were regarded as acceptable, whereas survival rates of ≥ 70% and ≥ 80% were classified as good and excellent tolerance, respectively [36].

### Assessment of LAB isolates’ cell surface hydrophobicity

Cell surface hydrophobicity is a key characteristic of LAB associated with their ability to adhere to the intestinal mucosa, which is considered a critical criterion for the selection of probiotics. The hydrophobicity of LAB isolates was assessed using the MATH method described by Rahman *et al*. [37]. Briefly, LAB cells were harvested by centrifugation at 8,000 × *g* for 10 min, and the optical density at 600 nm (OD_600_) of the bacterial suspension was measured and recorded as A^1^.A 3.0 mL aliquot of the suspension was then transferred into a clean test tube, and 1.0 mL of hydrocarbon (xylene: Cat. No. 9490-03; J. T. Baker, Avantor, United Kingdom) was added. The ratio of xylene to the bacterial suspension was 3:1 (v/v). The mixture was vigorously vortexed for 5 min and then left undisturbed for 10–15 min to facilitate phase separation. The aqueous (lower) phase was carefully collected, and its OD_600_ was measured and recorded as A_2_. The hydrophobicity index (HPBI) was calculated using the following equation:

HPBI (%) = [(A_1_– A_2_) / A_1_] × 100

According to commonly adopted criteria, probiotic candidates exhibiting hydrophobicity values of ≥ 40% are considered acceptable, whereas values of ≥ 60% and ≥ 70% are indicative of good and excellent hydrophobicity, respectively [38].

### Assessment of LAB isolate autoaggregation

The autoaggregation ability of the LAB isolates was evaluated following the method described by Kos *et al*. [39]. Briefly, the LAB isolates were grown in MRS broth at 37°C for 24 h under anaerobic conditions. Cultures were centrifuged at 8,000 × *g* for 10 min, and the bacterial pellets were washed twice with PBS (pH 7.4). The cell pellets were resuspended in PBS OD_600_ of approximately 0.6 (approximately 10^8^ CFU/mL). Bacterial suspensions were incubated at room temperature (25°C ± 2°C) without agitation. The optical density of the upper suspension was measured at 0 and 4 h. The autoaggregation was calculated using the following equation:

Autoaggregation (%) = [1 − (OD_600_ at 4 h / OD_600_ at 0 h)] × 100

According to commonly adopted criteria, probiotic candidates exhibiting autoaggregation values of ≥ 40% are considered acceptable, whereas values of ≥ 60% and ≥ 70% are indicative of good and excellent autoaggregation ability, respectively [38]. Higher autoaggregation percentages indicated a strong ability of the isolates to self-associate, a property considered advantageous for intestinal colonization and probiotic functionality.

All measurements were performed in 11 biological replicates (n = 11), and each biological replicate was analyzed in triplicate. The enumeration of LAB was conducted in accordance with ISO 15214:2015, and probiotic characteristics were evaluated following the guidelines recommended by the Food and Agriculture Organization/World Health Organization [40, 41].

### Safety assessment of the LAB isolates

#### The hemolytic activity test

The hemolytic activity of LAB isolates was assessed to evaluate their safety for potential use as probiotics. Each isolate was cultured in MRS broth at 37°C for 48 h and subsequently streaked onto Columbia agar (M144B; HiMedia, India) supplemented with 5% sheep red blood cells (RBC; Lot No. 240610; Clinical Diagnostics, Ltd., Thailand). The inoculated plates were incubated at 37°C for 48 h. Following incubation, the plates were examined for hemolysis zones. A clear zone surrounding the bacterial colonies, indicative of complete RBC lysis, was classified as β-hemolysis. *S. aureus* (ATCC 25923) strain exhibiting beta-hemolysis was used as a positive control. A greenish halo representing partial hemolysis was classified as α-hemolysis, while the absence of any discoloration or clearing around the colonies indicated γ-hemolysis, signifying a non-hemolytic phenotype [24].

#### Antibiotic susceptibility test

The antibiotic susceptibility of LAB isolates was evaluated using the disk diffusion method. LAB isolates that exhibited strong inhibitory activity against pathogenic bacteria were selected and cultured on MRS agar at 37°C for 48 h. Bacterial suspensions were then adjusted to a turbidity of 0.5 McFarland standard (approximately 10^8^ CFU/mL). The standardized suspensions were uniformly swabbed onto the surfaces of the MRS agar plates. Antibiotic disks containing ampicillin, chloramphenicol, enrofloxacin, tetracycline, and streptomycin (HiMedia, India) were placed on the inoculated agar. Plates were incubated at 37°C for 48 h. The diameters of the inhibition zones surrounding each disk were measured in millimeters following incubation. Antibiotic susceptibility testing was performed using the disk diffusion method in accordance with the guidelines of the Clinical and Laboratory Standards Institute. The isolates were classified as susceptible or resistant based on the established interpretive criteria for each antibiotic agent [42].

### Molecular identification using *16S rRNA* gene sequencing

Bacterial species were identified using molecular techniques targeting the *16S rRNA* gene. Genomic DNA was extracted from a bacterial suspension (approximately 10^9^ cells) using a commercial genomic DNA isolation kit (Bio-Helix, Taiwan) according to the manufacturer’s instructions. Total DNA quality was evaluated using a NanoDrop spectrophotometer (Maestrogen Inc., China), yielding an A260/280 absorbance ratio of 1.9, indicating high-quality DNA. Two microliters of the extracted DNA (10 ng/µL) served as a template for polymerase chain reaction (PCR) amplification using the universal bacterial primers 27F and 1492R [43]. The *16S rRNA* gene was amplified using these universal primers, generating an amplicon of approximately 1,500 bp. Briefly, 2 µL of 27F (10 ng/µL) and 2 µL of 1492R (10 ng/µL) in 25 µL of PCR master mix (Cat. No. KMM-101; Toyobo, Japan). The PCR cycling conditions were as follows: initial denaturation at 95°C for 2 min, followed by 35 cycles of denaturation at 95°C for 1 min, annealing at 55°C for 1 min, and extension at 72°C for 1 min, with a final extension at 72°C for 10 min. PCR products were purified using a MultiScreen filter plate (Millipore Corp., Bedford, MA, USA). The PRISM BigDye Terminator v3.1 Cycle Sequencing Kit (Applied Biosystems, Foster City, CA, USA) was used to perform sequencing reactions. The extension products were mixed with Hi-Di formamide (Applied Biosystems, Foster City, CA, USA), denatured at 95°C for 5 min, chilled on ice for 5 min, and analyzed using an ABI PRISM 3730XL DNA Analyzer (Applied Biosystems, Foster City, CA, USA).

### Phylogenetic analysis

Phylogenetic relationships were inferred using *16S rRNA* gene sequences with the maximum likelihood method and the Kimura 2-parameter model implemented in MEGA version 11 software (www.megasoftware. net). The best-fit nucleotide substitution model was selected based on the Akaike Information Criterion and Bayesian Information Criterion values implemented in MEGA, indicating that the K2P model provided the optimal fit for the dataset. The robustness of the tree topology was assessed using bootstrap analysis with 1,000 replicates. The percentage of replicate trees supporting each node is shown next to the corresponding branches. The Neighbor-Joining and BioNJ algorithms were used to automatically generate initial trees for the heuristic search based on a matrix of pairwise distances estimated by the Maximum Composite Likelihood approach, and the topology with the highest log-likelihood value was selected.

### Statistical analysis

Data normality was assessed using the Kolmogorov–Smirnov test before analysis. As the data were normally distributed, differences among groups were analyzed using one-way analysis of variance. When significant differences were detected, Duncan’s multiple range test was used to perform post hoc comparisons. Outlier detection was conducted using the Z-score method Z = (x − mean)/SD, and no outliers were identified. A p-value of <0.05 was considered statistically significant. All statistical analyses were performed using the Statistical Package for the Social Sciences software version 22 for Windows (SPSS Inc., Chicago, IL, USA). Data visualization and figure preparation were performed using Microsoft Excel and Microsoft Paint, respectively.

## RESULTS

### Isolation and preliminary phenotypic characterization of LAB

A total of 195 LAB isolates were recovered from fecal samples collected from 25 captive Asian elephants. All isolates were identified as Gram-positive, non-spore-forming bacteria and tested negative for catalase, oxidase, and indole activity. Morphological examination revealed that 133 isolates (68.20%) were Gram-positive rod-shaped bacteria, whereas the remaining 62 isolates (31.80%) exhibited a Gram-positive cocci-shaped morphology.

### Antimicrobial activity against pathogenic bacteria

Among the 195 LAB isolates obtained from elephant feces, 52 isolates (26.67%) exhibited inhibitory activity against all five tested pathogenic bacterial strains. A substantial proportion of isolates demonstrated antagonistic activity against at least one indicator organism. Specifically, 124 isolates (63.59%) inhibited *E. coli*, 115 (58.97%) inhibited *P. aeruginosa*, 117 (60.00%) inhibited *S*. Typhimurium, 130 (66.67%) inhibited *K. pneumoniae*, and 108 isolates (55.38%) inhibited *S. aureus*. These findings indicate the broad-spectrum antimicrobial potential of LAB isolates, with inhibition zone diameters ≥12 mm.

**Figure 1:**
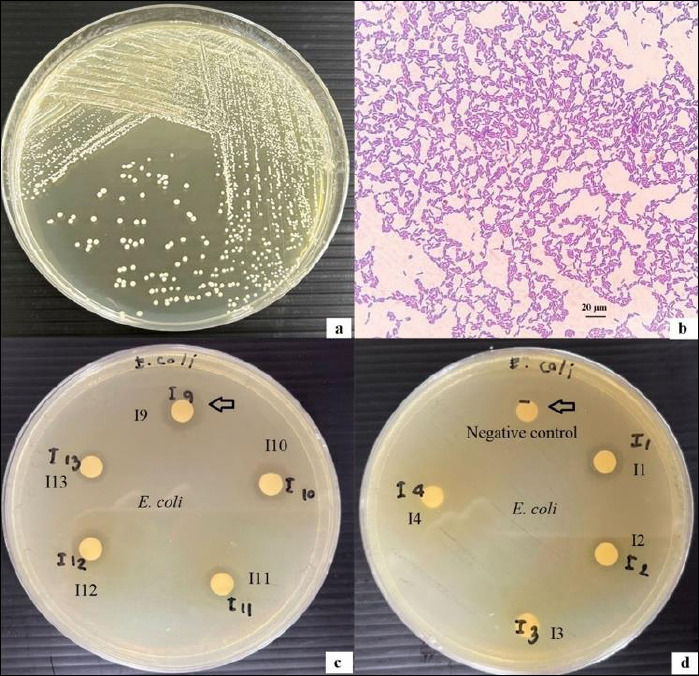
(a) Colony morphology of isolate I9; (b) Gram-stained micrograph of isolate I9 showing Gram-positive rod-shaped bacteria under light microscopy (100×); (c) disk diffusion assay illustrating the antimicrobial activity of the cell-free supernatant from isolate I9 cultured in de Man, Rogosa, and Sharpe (MRS) broth against *Escherichia coli*, with the largest inhibition zone indicated by an arrow; (d) negative control (sterile MRS broth without lactic acid bacteria) showing no inhibition zone (arrow). Inhibition zone diameters include the 6-mm paper disc. Scale bar = 20 µm.

The 52 isolates that inhibited all five pathogenic bacteria were selected for further evaluation of probiotic properties. Among these, 11 isolates (I1, I9, I12, I56, I90, I91, I115, I131, I145, I146, and I182) exhibited favorable characteristics, including acid and bile salt tolerance, cell surface hydrophobicity, and autoaggregation capacity, all exceeding 50%. Further analysis showed that isolates I9, I12, I115, and I145 exhibited significantly higher inhibitory activity (p < 0.05) against *S*. Typhimurium, while isolate I9 demonstrated the strongest inhibition of *E. coli* (Figure 1). The antimicrobial activity and probiotic properties of the selected isolates are summarized in Table 1.

**Table 1:** Antimicrobial activity of 11 lactic acid bacteria isolates against selected pathogenic microorganisms, as determined by the disk diffusion assay.

Isolate	*Escherichia coli*	*Salmonella* Typhimurium	*Pseudomonas aeruginosa*	*Klebsiella pneumoniae*	*Staphylococcus aureus*
I1	12.01 ± 0.01^c^	12.02 ± 0.02^cd^	12.31 ± 0.03^de^	12.01 ± 0.01^e^	12.02 ± 0.03^b^
I9	13.20 ± 0.01^a^	12.09 ± 0.02^a^	12.64 ± 0.01^b^	12.86 ± 0.10^a^	12.22 ± 0.03^a^
I12	12.00 ± 0.00^c^	12.10 ± 0.01^a^	12.31 ± 0.03^de^	12.04 ± 0.06^de^	12.07 ± 0.02^b^
I56	12.05 ± 0.03^b^	12.08 ± 0.01^ab^	12.60 ± 0.02^c^	12.58 ± 0.04^b^	12.19 ± 0.01^a^
I90	12.01 ± 0.01^c^	12.05 ± 0.03^bc^	12.38 ± 0.02^d^	12.01 ± 0.02^e^	12.01 ± 0.01^b^
I91	12.03 ± 0.03^bc^	12.02 ± 0.03^cd^	12.38 ± 0.03^d^	12.00 ± 0.01^e^	12.09 ± 0.13^b^
I115	12.01 ± 0.01^c^	12.09 ± 0.02^a^	12.26 ± 0.03^e^	12.12 ± 0.11^cd^	12.09 ± 0.05^b^
I131	12.00 ± 0.01^c^	12.02 ± 0.02^cd^	12.26 ± 0.02^e^	12.11 ± 0.02^cd^	12.04 ± 0.05^b^
I145	12.05 ± 0.02^b^	12.11 ± 0.04^a^	12.88 ± 0.02^a^	12.56 ± 0.02^b^	12.19 ± 0.02^a^
I146	12.01 ± 0.02^c^	12.00 ± 0.00^d^	12.26 ± 0.01^e^	12.04 ± 0.04^de^	12.20 ± 0.03^a^
I182	12.00 ± 0.01^c^	12.00 ± 0.01^d^	12.31 ± 0.04^de^	12.15±0.01^c^	12.06±0.02^b^

(a) Colony morphology of isolate I9; (b) Gram-stained micrograph of isolate I9 showing Gram-positive rod-shaped bacteria under light microscopy (100×); (c) disk diffusion assay illustrating the antimicrobial activity of the cell-free supernatant from isolate I9 cultured in de Man, Rogosa, and Sharpe (MRS) broth against *Escherichia coli*, with the largest inhibition zone indicated by an arrow; (d) negative control (sterile MRS broth without lactic acid bacteria) showing no inhibition zone (arrow). Inhibition zone diameters include the 6-mm paper disc. Scale bar = 20 µm.

Results are presented as mean inhibition zone diameters (mm) ± standard deviation from three independent replicates (n = 11). Mean values within the same column followed by different superscript letters indicate statistically significant differences (p < 0.05). Inhibition zone diameters include the diameter of the paper disc (6 mm). *E. coli*, *S*. Typhimurium, *P. aeruginosa*, *K. pneumoniae*, and *S. aureus* were used as indicator pathogenic microorganisms.

### Acid and bile salt tolerance of LAB isolates

The acid and bile salt tolerance of the 11 selected LAB isolates is summarized in Table 2. All isolates exhibited survival rates greater than 50% under acidic conditions (pH 3.0) and in the presence of 1% bile salts, indicating physiological resilience compatible with probiotic application. Isolate I9 showed the highest tolerance to both acid and bile salt exposure. Notably, four isolates (I1, I9, I56, and I145) exhibited acid tolerance rates exceeding 90%, while two isolates (I9 and I145) demonstrated bile salt tolerance rates above 95%.

**Table 2:** Acid and bile salt tolerance of 11 lactic acid bacteria isolates.

Isolate	Acid tolerance (%)	Bile salt tolerance (%)
I1	91.17 ± 1.04^ab^	93.33 ± 2.89^b^
I9	91.67 ± 0.58^a^	98.15 ± 3.21^a^
I12	85.43 ± 0.04^c^	82.39 ± 0.34^c^
I56	91.13 ± 1.08^ab^	91.09 ± 3.86^b^
I90	80.42 ± 0.72^f^	93.49 ± 2.61^b^
I91	74.67 ± 0.58^g^	91.50 ± 0.87^b^
I115	80.67 ± 0.58^f^	80.26 ± 0.44^cd^
I131	83.83 ± 0.29^d^	75.17 ± 0.29^e^
I145	90.23 ± 0.23^b^	95.16 ± 4.76^ab^
I146	82.50 ± 0.87^e^	91.83 ± 0.76^b^
I182	86.52 ± 0.50^c^	77.07 ± 0.12^de^

Values are presented as mean ± standard deviation from three independent replicates (n = 11). Mean values within the same column followed by different superscript letters indicate statistically significant differences (p < 0.05). Acid tolerance was assessed at pH 3.0, and bile salt tolerance was evaluated using 1% bile salts. Survival percentages were calculated relative to initial viable counts.

### Cell surface hydrophobicity of LAB isolates

**Figure 2:**
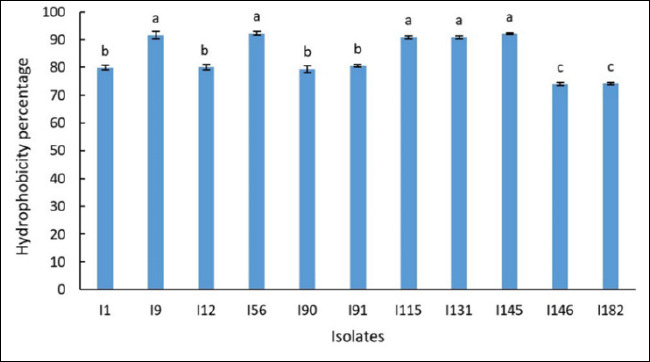
Cell surface hydrophobicity of 11 lactic acid bacteria isolates. Values are expressed as mean ± standard deviation from three independent replicates (n = 11). Different superscript letters indicate statistically significant differences (p < 0.05).

As shown in Figure 2, isolates I9, I56, I115, I131, and I145 exhibited the highest levels of cell surface hydrophobicity among all tested strains. In contrast, isolates I146 and I182 displayed the lowest hydrophobicity values.

### Autoaggregation ability of LAB isolates

**Figure 3:**
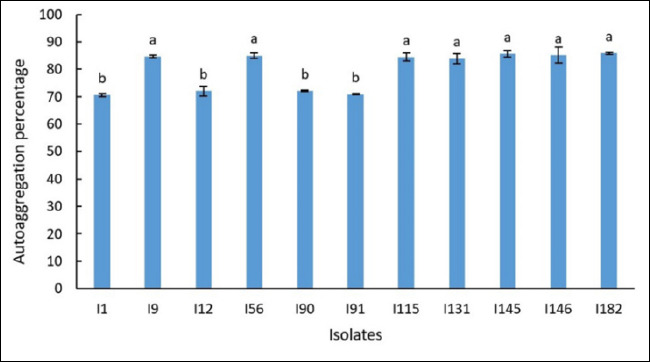
Autoaggregation capacity of 11 lactic acid bacteria isolates. Values are presented as mean ± standard deviation from three independent replicates (n = 11). Different superscript letters indicate statistically significant differences (p < 0.05).

High autoaggregation capacity was observed in isolates I9, I56, I115, I131, I145, I146, and I182 (Figure 3), indicating a strong ability for cell-to-cell adhesion, a trait associated with enhanced colonization potential within the host gastrointestinal tract. Conversely, isolates I1, I12, I90, and I91 exhibited comparatively lower autoaggregation values.

### Safety assessment of LAB isolates

All 11 selected LAB isolates were susceptible to five commonly used antibiotics, namely ampicillin, chloramphenicol, streptomycin, tetracycline, and enrofloxacin (Table 3). In addition, hemolysis assays performed on Columbia agar supplemented with 5% sheep RBC confirmed that all isolates exhibited non-hemolytic activity, corresponding to gamma-hemolysis.

**Table 3:** Antibiotic susceptibility profile of selected isolates.

Isolate	Ampicillin	Chloramphenicol	Enrofloxacin	Tetracycline	Streptomycin
I1	S	S	S	S	S
I9	S	S	S	S	S
I12	S	S	S	S	S
I56	S	S	S	S	S
I90	S	S	S	S	S
I91	S	S	S	S	S
I115	S	S	S	S	S
I131	S	S	S	S	S
I145	S	S	S	S	S
I146	S	S	S	S	S
I182	S	S	S	S	S

Isolates were classified as susceptible (S) when inhibition zone diameters were ≥17.5 mm for ampicillin, chloramphenicol, streptomycin, and tetracycline, and ≥18.0 mm for enrofloxacin. Resistance (R) was defined as inhibition zone diameters ≤14.5 mm for ampicillin, chloramphenicol, streptomycin, and tetracycline, and ≤14.0 mm for enrofloxacin.

### Molecular identification and phylogenetic analysis

**Figure 4:**
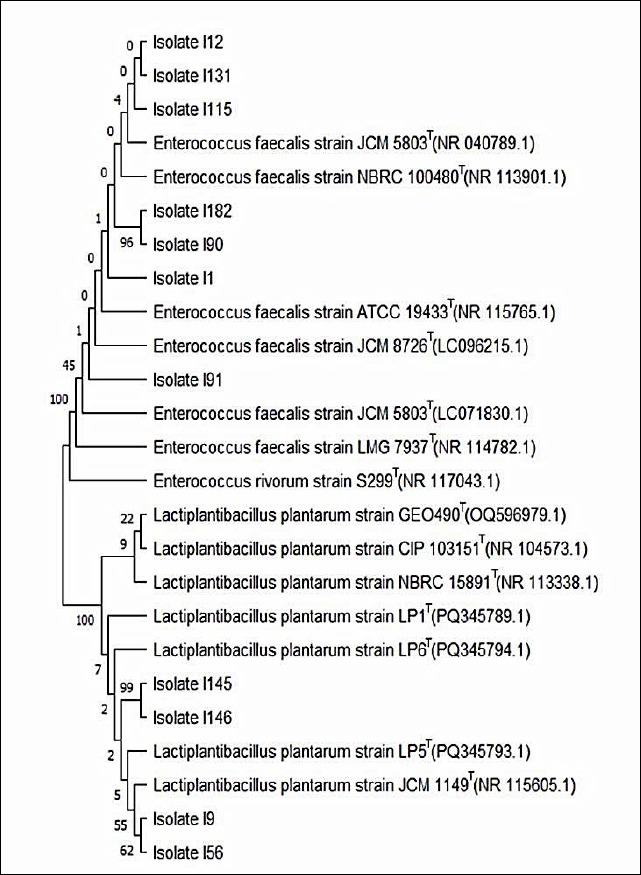
Phylogenetic tree of 11 lactic acid bacteria isolates exhibiting probiotic characteristics, constructed using *16S rRNA* gene sequences and the maximum likelihood method with the Kimura two-parameter model implemented in MEGA version 11 software. Tree topology robustness was evaluated by bootstrap analysis based on 1,000 replicates. The tree with the highest log-likelihood value is presented. Bootstrap support values, expressed as percentages, are shown next to the corresponding branches and indicate the frequency with which associated taxa clustered together among replicate trees. Initial trees for the heuristic search were generated using the Neighbor-Joining and BioNJ algorithms based on a matrix of pairwise distances estimated by the Maximum Composite Likelihood approach, and the topology with the highest log-likelihood value was selected.

The 11 selected LAB isolates were subjected to molecular identification using *16S rRNA* gene sequencing followed by phylogenetic analysis (Figure 4). Sequence alignment results identified four isolates as *L. plantarum*, with sequence identity values ranging from 99.54% to 99.87%. The remaining seven isolates were identified as *Enterococcus faecalis*, with sequence identities ranging from 99.58% to 99.80%. Detailed molecular identification data are presented in Table 4. This study represents the first molecularly validated identification of *L. plantarum* strains isolated from captive Asian elephants based on full-length *16S rRNA* gene sequencing and phylogenetic confirmation.

**Table 4:** Molecular identification of 11 lactic acid bacteria isolates based on *16S rRNA* gene sequencing.

Isolate	Amplicon size (bp)	Closest reference strain (accession number)	Identity (%)
I1	1452	*Enterococcus faecalis* strain NBRC 100480 (NR_113901.1)	99.80
I9	1519	*Lactiplantibacillus plantarum* strain JCM 1149 (NR_115605.1)	99.87
I12	1522	*Enterococcus faecalis* strain JCM 5803 (NR_040789.1)	99.79
I56	1519	*Lactiplantibacillus plantarum* strain JCM 1149 (NR_115605.1)	99.54
I90	1426	*Enterococcus faecalis* strain NBRC 100480 (NR_113901.1)	99.58
I91	1500	*Enterococcus faecalis* strain JCM 8726 (LC096215.1)	99.65
I115	1517	*Enterococcus faecalis* strain JCM 5803 (NR_040789.1)	99.67
I131	1517	*Enterococcus faecalis* strain JCM 5803 (NR_040789.1)	99.80
I145	1488	*Lactiplantibacillus plantarum* strain LP5 (PQ345793.1)	99.57
I146	1488	*Lactiplantibacillus plantarum* strain LP5 (PQ345793.1)	99.66
I182	1453	*Enterococcus faecalis* strain NBRC 100480 (NR_113901.1)	99.72

Amplicon size refers to the length of the amplified *16S rRNA* gene fragment. Closest reference strains were identified by sequence alignment against the GenBank database, and accession numbers are provided in parentheses. Identity (%) indicates the percentage sequence similarity between each isolate and its closest reference strain.

Phylogenetic tree of 11 lactic acid bacteria isolates exhibiting probiotic characteristics, constructed using *16S rRNA* gene sequences and the maximum likelihood method with the Kimura two-parameter model implemented in MEGA version 11 software. Tree topology robustness was evaluated by bootstrap analysis based on 1,000 replicates. The tree with the highest log-likelihood value is presented. Bootstrap support values, expressed as percentages, are shown next to the corresponding branches and indicate the frequency with which associated taxa clustered together among replicate trees. Initial trees for the heuristic search were generated using the Neighbor-Joining and BioNJ algorithms based on a matrix of pairwise distances estimated by the Maximum Composite Likelihood approach, and the topology with the highest log-likelihood value was selected.

## DISCUSSION

### Overview of isolation and antimicrobial potential of LAB

This study successfully isolated 195 LAB strains from fecal samples of 25 healthy Asian elephants. To the best of our knowledge, this is the first study to conduct a comprehensive, multidimensional evaluation of elephant-derived LAB by integrating antimicrobial activity, acid and bile tolerance, cell surface hydrophobicity, autoaggregation, antibiotic susceptibility, and hemolytic safety testing. Among these, 52 isolates (26.67%) demonstrated antimicrobial activity against five clinically pathogenic bacteria, namely *E. coli*, *P. aeruginosa*, *S*. Typhimurium, *K. pneumoniae*, and *S. aureus*, which are common causative agents of gastrointestinal and opportunistic infections in elephants, other animals, and humans [27–31].

Isolate I9 showed the highest inhibition of *E. coli* (Figure 1) and *K. pneumoniae*. Isolates I9, I12, I115, and I145 exhibited significantly higher inhibition of *S*. Typhimurium, whereas isolate I145 showed the strongest inhibition of *P. aeruginosa*. In addition, isolates I9, I56, I145, and I146 exhibited significant inhibition of *S. aureus*. This study represents the first report demonstrating strong broad-spectrum antimicrobial activity of elephant-derived *L. plantarum* against clinically relevant enteric and opportunistic pathogens. These findings highlight strain-specific variation in antimicrobial efficacy among the isolates, consistent with previous reports [18].

### Selection of indicator pathogens and antimicrobial mechanisms

The selection of *E. coli* (ATCC 25922), *S*. Typhimurium (ATCC 13311), *P. aeruginosa* (ATCC 27853), *S. aureus* (ATCC 25923), and *K. pneumoniae* (ATCC 700603) as indicator pathogens followed internationally accepted probiotic screening strategies, ensuring methodological robustness and enabling comparison with existing studies. As environmentally ubiquitous bacteria, their inclusion provides ecologically relevant preliminary evidence of the capacity of probiotic strains to inhibit common enteric and opportunistic pathogens encountered by elephants through dietary and environmental exposure.

The inhibition zones produced by the cell-free supernatant of LAB isolates ranged from 11 to 13 mm, classifying them as strong antimicrobial activity according to established criteria [32]. These findings are consistent with previous studies in which *L. plantarum* isolated from swine feces inhibited swine pathogens [44], and *L. plantarum* strains derived from sheep feces suppressed foodborne pathogens [45]. In addition, LAB species such as *Lactobacillus pentosus*, *Lactococcus garvieae*, and *Enterococcus hirae* have been isolated from wild elephant feces in Thailand [46]. The antimicrobial activity of probiotics is attributed to the production of bioactive compounds, including bacteriocins, lactic acid, acetic acid, propionic acid, ethanol, and antimicrobial peptides [47]. Variations in antimicrobial efficacy among isolates may reflect differences in microbial species and growth conditions that influence the type and quantity of antimicrobial compounds produced [48].

### Acid and bile salt tolerance of elephant-derived LAB

Acid and bile salt tolerance are critical attributes for probiotic bacteria to survive gastrointestinal transit and establish persistence in the host. In this study, acid tolerance was assessed at pH 3.0, reflecting the typical gastric pH range observed in elephants [49]. Previous studies have shown that the robust cell wall structure of Lactobacilli, composed primarily of thick peptidoglycan and teichoic acids, contributes to resistance under acidic conditions [50].

Bile salts exert antimicrobial effects by disrupting bacterial phospholipid bilayers and cell membranes, leading to cell lysis [51], and higher bile salt concentrations are associated with increased bacterial mortality [52]. The 1% bile salt concentration used in this study represents a physiologically relevant level in the intestinal tract of animals [53]. Exopolysaccharide production by *L. plantarum* has been reported to enhance resistance to acidic and bile stress conditions [54]. All 11 LAB isolates demonstrated survival rates exceeding 50% under these stress conditions, with isolate I9 showing the highest tolerance to both acid and bile salts, indicating strong potential for gastrointestinal survival.

### Adhesion-related properties: hydrophobicity and autoaggregation

Cell surface hydrophobicity and autoaggregation are key attributes contributing to bacterial adhesion and intestinal mucosal colonization. High hydrophobicity is generally associated with increased adherence to epithelial cells and is influenced by bacterial cell envelope components, including lipoteichoic acids, teichoic acids, S-layer proteins [55], and mannose-specific lectins [56]. Together with autoaggregation, these properties contribute to microbial homeostasis and competitive exclusion of pathogenic microorganisms [57].

Previous studies have shown that *L. plantarum*, *L. rhamnosus*, *L. acidophilus* [58], and *Lactobacillus salivarius* [59] can initiate autoaggregation within minutes to several hours, depending on environmental and growth conditions. Hydrophobicity and autoaggregation are strain-specific traits that vary among *Lactobacillus* isolates [60]. In this study, isolates I9, I56, I115, I131, and I145 exhibited significantly higher hydrophobicity and autoaggregation than other tested strains, indicating enhanced mucosal adhesion and colonization potential. The *L. plantarum* strains were isolated directly from the elephant gut ecosystem and exhibited traits consistent with adaptation to hindgut fermentation physiology, supporting the concept of host-specific probiotics.

### Safety profile of elephant-derived LAB

Antibiotic susceptibility and hemolytic activity are critical parameters for probiotic safety assessment. Hemolytic activity facilitates host tissue invasion by pathogenic bacteria; therefore, the absence of hemolysis is a key safety requirement [61]. All 11 LAB isolates were susceptible to ampicillin, chloramphenicol, streptomycin, tetracycline, and enrofloxacin, suggesting a low risk of transferable antibiotic resistance.

Furthermore, none of the isolates exhibited hemolytic activity when cultured on blood agar supplemented with sheep RBC, and all were classified as gamma-hemolytic. These findings provide the first verified safety profile of elephant-derived LAB, supporting their suitability for probiotic development in animals and humans.

### Molecular identification and relevance of dominant LAB species

Molecular identification based on *16S rRNA* gene sequencing revealed that four isolates belonged to *L. plantarum* and seven isolates were identified as *E. faecalis*. Both species are commonly present in the gastrointestinal tract and have been widely studied for probiotic potential. *L. plantarum* is recognized for its safety and efficacy as a probiotic in humans and animals [55]. Although *E. faecalis* is considered a commensal organism, its opportunistic pathogenic potential necessitates careful strain-level safety evaluation. Non-hemolytic and antibiotic-sensitive *E. faecalis* strains, such as those identified in this study, may be considered acceptable probiotic candidates [62]. However, concerns regarding virulence factors and antimicrobial resistance limit broader application of Enterococcus-based probiotics, further supporting *L. plantarum* as the primary candidate for continued investigation.

### Identification of elite probiotic candidates and broader implications

Among the identified isolates, I9, I56, I115, I131, and I145 exhibited significantly higher hydrophobicity and autoaggregation. Isolates I9, I56, and I145 demonstrated the most promising probiotic potential overall, based on superior performance across antimicrobial activity, acid and bile salt tolerance, hydrophobicity, and autoaggregation. All three isolates were identified as *L. plantarum* based on *16S rRNA* gene analysis. This study represents the first report identifying elite LAB strains from elephants with consistently strong probiotic profiles.

The probiotic potential of *L. plantarum* has also been reported in other megaherbivores, including rhinoceros (*Rhinoceros* spp.) [63], hippopotamus (*Hippopotamus amphibius*) [64], and giraffes (*Giraffa camelopardalis*) [65]. These findings indicate that *L. plantarum* is a recurrent component of the gastrointestinal microbiota of large herbivores. Although *L. plantarum* is not a primary producer of short-chain fatty acids, LAB can support short-chain fatty acid metabolism through cross-feeding interactions [66–69]. The strong tolerance, hydrophobicity, and autoaggregation observed in elephant-derived strains suggest persistence and functional relevance in the elephant hindgut. Host-derived strains may offer improved adaptation compared with non-host commercial probiotics, warranting further *in vivo* validation. From a One Health perspective, host-specific probiotics for elephants may reduce antibiotic use, limit antimicrobial resistance selection, and decrease pathogen dissemination across animals, the environment, and humans [70].

## CONCLUSION

This study demonstrated that fecal samples from healthy Asian elephants harbor a diverse population of LAB with promising probiotic characteristics. Among 195 LAB isolates, 52 exhibited broad-spectrum antimicrobial activity against clinically relevant pathogens, including *E. coli*, *P. aeruginosa*, *S*. Typhimurium, *K. pneumoniae*, and *S. aureus*. Eleven isolates fulfilled key probiotic selection criteria, showing strong antimicrobial activity, survival under acidic and bile salt stress, high cell surface hydrophobicity, and autoaggregation capacity. Molecular identification based on *16S rRNA* gene sequencing revealed that the most robust isolates belonged predominantly to *L. plantarum*, with selected E. faecalis strains also exhibiting acceptable safety profiles.

The identification of elephant-derived LAB, particularly *L. plantarum*, provides a scientifically grounded basis for the development of host-adapted probiotic formulations for elephants. Such probiotics may support gastrointestinal health, enhance resilience against enteric pathogens, and mitigate dysbiosis in captive or stressed animals. From a management perspective, the use of host-specific probiotics could reduce reliance on antibiotics, thereby contributing to antimicrobial stewardship and improved health outcomes in elephant care and conservation programs.

A major strength of this study lies in its comprehensive, multidimensional screening strategy, integrating antimicrobial efficacy, functional probiotic traits, safety assessment, and molecular confirmation within a single experimental framework. The use of standardized *in vitro* assays and full-length *16S rRNA* gene sequencing ensured methodological rigor and reproducibility. Importantly, this work provides the first molecularly validated evidence of elite elephant-derived *L. plantarum* strains with consistent probiotic potential.

Despite these strengths, the study was limited to *in vitro* evaluations and did not include *in vivo* validation in Asian elephants. Certain functional attributes, such as bile salt hydrolase activity, epithelial adhesion under dynamic gut conditions, and direct quantification of short-chain fatty acid production, were not assessed. In addition, the absence of a certified reference probiotic strain as a positive control represents a methodological constraint.

Future research should prioritize *in vivo* validation of selected *L. plantarum* strains in Asian elephants to assess gastrointestinal colonization, safety, and health outcomes. Studies evaluating strain-specific dosage, formulation stability, and delivery strategies are warranted. Comparative investigations involving captive and wild Asian elephants would further clarify host–microbiota interactions. From a One Health perspective, exploring the role of host-derived probiotics in reducing pathogen shedding and antimicrobial resistance dissemination represents an important and timely research direction.

Overall, this study establishes elephant-derived *L. plantarum* as a promising host-specific probiotic candidate with strong functional and safety profiles. The findings advance current understanding of elephant gut microbiota and provide a foundational framework for microbiome-based interventions aimed at improving elephant health, supporting sustainable management practices, and contributing to broader One Health objectives.

## DATA AVAILABILITY

All the generated data are included in the manuscript.

## AUTHORS’ CONTRIBUTIONS

MS and WB: Conceived and designed the study. RS and PM: Fieldwork and sample collection. MS, SM, and SC: Laboratory experiments. MS, WB, WS, and DC: Data analysis and interpretation. MS: The manuscript was drafted. WB: The manuscript has been revised. All authors have read, reviewed, and approved the final version of the manuscript.

## COMPETING INTERESTS

The authors declare that they have no competing interests.

## PUBLISHER’S NOTE

Veterinary World remains neutral with regard to jurisdictional claims in the published institutional affiliations.
